# Quantitative Analysis of *BRCA1* and *BRCA2* Germline Splicing Variants Using a Novel RNA-Massively Parallel Sequencing Assay

**DOI:** 10.3389/fonc.2018.00286

**Published:** 2018-07-27

**Authors:** Suzette Farber-Katz, Vickie Hsuan, Sitao Wu, Tyler Landrith, Huy Vuong, Dong Xu, Bing Li, Jayne Hoo, Stephanie Lam, Sarah Nashed, Deborah Toppmeyer, Phillip Gray, Ginger Haynes, Hsiao-Mei Lu, Aaron Elliott, Brigette Tippin Davis, Rachid Karam

**Affiliations:** ^1^Translational Genomics Laboratory, Ambry Genetics, Aliso Viejo, CA, United States; ^2^Department of Bioinformatics, Ambry Genetics, Aliso Viejo, CA, United States; ^3^Department of Research and Development, Ambry Genetics, Aliso Viejo, CA, United States; ^4^Rutgers Cancer Institute of New Jersey, Rutgers, The State University of New Jersey, New Brunswick, NJ, United States

**Keywords:** genetic testing, hereditary breast and ovarian cancer, HBOC, *BRCA1*, *BRCA2*, RNA, Splicing, NGS

## Abstract

Clinical genetic testing for hereditary breast and ovarian cancer (HBOC) is becoming widespread. However, the interpretation of variants of unknown significance (VUS) in HBOC genes, such as the clinically actionable genes *BRCA1* and *BRCA2*, remain a challenge. Among the variants that are frequently classified as VUS are those with unclear effects on splicing. In order to address this issue we developed a high-throughput RNA-massively parallel sequencing assay—*CloneSeq*—capable to perform quantitative and qualitative analysis of transcripts in cell lines and HBOC patients. This assay is based on cloning of RT-PCR products followed by massive parallel sequencing of the cloned transcripts. To validate this assay we compared it to the RNA splicing assays recommended by members of the ENIGMA (Evidence-based Network for the Interpretation of Germline Mutant Alleles) consortium. This comparison was performed using well-characterized lymphoblastoid cell lines (LCLs) generated from carriers of the *BRCA1* or *BRCA2* germline variants that have been previously described to be associated with splicing defects. CloneSeq was able to replicate the ENIGMA results, in addition to providing quantitative characterization of *BRCA1* and *BRCA2* germline splicing alterations in a high-throughput fashion. Furthermore, CloneSeq was used to analyze blood samples obtained from carriers of *BRCA1* or *BRCA2* germline sequence variants, including the novel uncharacterized alteration *BRCA1* c.5152+5G>T, which was identified in a HBOC family. CloneSeq provided a high-resolution picture of all the transcripts induced by *BRCA1* c.5152+5G>T, indicating it results in significant levels of exon skipping. This analysis proved to be important for the classification of *BRCA1* c.5152+5G>T as a clinically actionable likely pathogenic variant. Reclassifications such as these are fundamental in order to offer preventive measures, targeted treatment, and pre-symptomatic screening to the correct individuals.

## Introduction

Correct interpretation of genomic sequence variants, and subsequent classification of variants as benign or pathogenic, is of utmost importance to patient management, especially in clinically actionable genes such as the breast and ovarian cancer susceptibility genes *BRCA1* and *BRCA2* (OMIM 113705 and 600185, respectively). Variant interpretation is based on multiple lines of evidence (1), including molecular and functional analysis, highlighting the urgent need to develop and implement high-throughput functional assays for variant classification (2).

Genomic sequence variants in *BRCA1* and *BRCA2* have the potential to alter normal splicing of these genes (3). In fact, many alterations in *BRCA1* and *BRCA2* have been shown to be clinically significant by RNA studies and multifactorial likelihood analyses that combine bioinformatics, pathologic, and clinical data (4–6). These variants include those that affect splicing by abolishing or weakening the canonical splice sites at intron-exon boundaries, by creating a novel or activating a cryptic splice site, or by disrupting enhancer or silencer splicing regulatory sequences (7).

Recommendations for mRNA analysis best practice in clinical testing were published by the Evidence-based Network for the Interpretation of Germline Mutant Alleles (ENIGMA) (8), a consortium established in 2009 with the purpose of sharing data, methods, and resources to facilitate the classification of sequence variants in hereditary breast and ovarian cancer (HBOC) genes (9). ENIGMA recommends the use of RT-PCR and digital or capillary electrophoresis to detect abnormal transcripts based on the length of the product observed, followed by cloning and Sanger sequencing to characterize the sequence of these transcripts (8). However, the consortium notes that evaluation of splicing results for variant carriers can be complicated by the detection of normal alternatively spliced transcripts (8). Both *BRCA1* and *BRCA2* genes are known to undergo alternative splicing, and clinical and functional data indicate that alternatively spliced transcripts may retain function (10, 11). Two fundamental issues in determining the functional significance of normal and abnormal spliced transcripts are whether a transcript is out-of-frame, and therefore predicted to be targeted to degradation by the nonsense-mediated RNA decay (NMD) pathway (12), and the level at which these transcripts are expressed (13). Therefore, a combination of qualitative and quantitative analysis is needed to provide proper characterization of splice variations, and to establish the clinical significance of these specific alterations.

**Figure 1:**
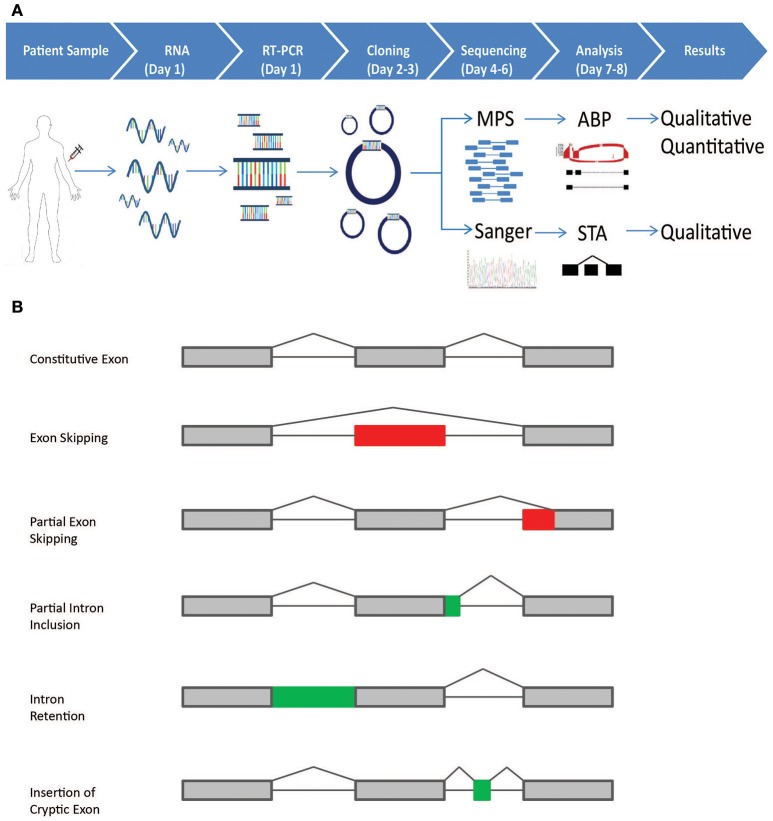
Schematic representation of the CloneSeq protocol and splicing events detected by the bioinformatics pipeline**. (A)** Blood from normal healthy controls and patients participating in the Ambry Genetics Family Studies program was drawn in PAXgene Blood RNA Tubes. RT-PCR was performed following ENIGMA recommendations. RT-PCR products were cloned into pGEM-T Easy and transformed into bacteria. For CloneSeq, all colonies on a plate were scraped and suspended in PBS. Plasmids were extracted, CloneSeq libraries were constructed, and Massively Parallel Sequencing (MPS) was performed, which generated 2 × 250 paired-end reads. The mapped reads are then analyzed by the customized Ambry Bioinformatic Pipeline (ABP) software to generate qualitative and quantitative data for splicing events, including exon skipping, alternative 5′ donor site, alternative 3′ acceptor site, and intron retention. We confirmed CloneSeq results by comparing the data with ENIGMA-recommended assays, in which several individual positive colonies were picked, amplified by rolling-circle amplification, and Sanger sequenced. Single-transcript alignment (STA) was performed to characterize the transcripts' sequences. **(B)** The five types of alternative splicing events, as described by Diederichs et al. that can be detected by the ABP: (1) exon skipping; (2) partial exon skipping (as a result of the usage of alternative exonic donor or acceptor site); (3) partial intron inclusion (as a result of the usage of alternative intronic donor or acceptor site);(4) intron retention;(5) insertion of cryptic exons.

With these in mind, we developed CloneSeq, a high-throughput RNA-based massively parallel sequencing (MPS) technique designed to perform quantitative and qualitative characterization of splicing alterations in a time-frame necessary for clinical testing. Here we describe this technique and perform a comparison of CloneSeq with the techniques recommended by the ENIGMA consortium (8). We performed this comparison using four well-characterized lymphoblastoid cell lines (LCLs) generated from carriers of the *BRCA1* or *BRCA2* germline variants *BRCA1* c.5467+5G>T, *BRCA1* c.135-1G>T, *BRCA2* c.8632+1G>A, or *BRCA2* c.9501+3A>T. These variants have been previously described and are known to be associated with splicing defects (8). Additionally, we used a similar strategy to analyze blood samples obtained from carriers of *BRCA1* or *BRCA2* germline sequence variants (Figure 1A), including the uncharacterized alteration *BRCA1* c.5152+5G>T identified in a novel HBOC family.

## Material and methods

### Samples

This study was approved and carried out in accordance with the recommendations of the Western Institutional Review Board (WIRB). All subjects gave written informed consent in accordance with the Declaration of Helsinki. Blood from normal healthy controls or patients participating in the Ambry Genetics Family Studies program was drawn in PAXgene Blood RNA Tubes and stored according to the manufacturer's recommendations (PreAnalytiX, Hombrechtikon, Switzerland). RNA was extracted using the PAXgene Blood RNA Kit according to the recommended protocol (PreAnalytiX). Informed consent was obtained from all participants. Breast RNA was purchased from Amsbio (Lake Forest, CA, USA) and BioChain (Newark, CA, USA). RNA quality was determined using the RIN number calculated by the TapeStation 2200 with RNA ScreenTape or High Sensitivity RNA ScreenTape (Agilent, Santa Clara, CA, USA).

### Cell lines

Lymphoblastoid cell lines (LCLs) were obtained from the Kathleen Cuningham Consortium for Research into Familial Breast Cancer (kConFab, Melbourne, Australia) from 4 carriers of *BRCA1* or *BRCA2* variants and 2 controls. Genotypes were verified by Sanger sequencing. LCLs were maintained according to the recommendations of kConFab. Inhibition of nonsense-mediated decay (NMD) was performed using puromycin (300 μg/ml) or cycloheximide (100 μg/ml) for 4 h, as previously described (8, 14).

### RNA analysis

cDNA was generated using the SuperScript IV First-Strand Synthesis System (Thermo Fisher Scientific, Chino, CA, USA). PCR was performed using either Platinum SuperFi PCR Master Mix (Thermo Fisher Scientific) or HotStarTaq Master Mix (Qiagen, Valencia, CA, USA) as previously described (8).

PCR products were analyzed using digital electrophoresis with D1000 ScreenTape and Reagents on the TapeStation 2200 (Agilent). Capillary electrophoresis (CE) was performed on an ABI 3730xl using MapMarker1000 as a standard (BioVentures, Murfreesboro, TN, USA). Primers were tagged at the 5′ end with FAM or HEX for detection by CE. CE analysis was performed with GeneMapper software (Thermo Fisher Scientific).

PCR products were cloned into pGEM-T Easy and transformed into bacteria according to the manufacturer's recommended protocol (Promega, Fitchburg, WI, USA). Individual white colonies were picked, amplified by rolling-circle replication, and Sanger sequenced by Genewiz (La Jolla, CA, USA).

For CloneSeq, cDNA, PCR, and cloning were performed as described above. All colonies on a plate were scraped and suspended in PBS. Plasmids were extracted with the GeneJET Plasmid Miniprep kit (Thermo Fisher Scientific). CloneSeq libraries were constructed according to the protocol outlined by KAPA Biosystems (Wilmington, MA, USA) using the Hyper Prep kit. Briefly, DNA was sheared to an average size of 250–350 bp using sonication (Covaris, Woburn, MA, USA). DNA fragment ends were repaired and phosphorylated. An “A” base was added to the 3′ end of the blunted fragments, followed by ligation of single-indexed adapters via T-A mediated ligation. The size and concentration of the DNA library were determined using the TapeStation 2200. Massively Parallel Sequencing (MPS) was performed on an Illumina MiSeq, which generated 2 × 250 paired-end reads. Sequencing reads were aligned to the hg19 reference genome and analyzed using Ambry's Bioinformatic Pipeline (see below).

For whole transcriptome RNA-Seq, globin mRNA and ribosomal RNA were depleted using the Globin-Zero Gold rRNA removal kit (Illumina, San Diego, CA, USA). After depletion, RNA was fragmented and single-indexed cDNA libraries were generated using an RNA Hyper Prep kit (KAPA Biosystems). Quality control was performed using the TapeStation 2200. Libraries were checked for average fragment size, concentration, and the presence of spurious peaks such as adapter dimers. Concentration was confirmed using a Qubit Fluorometer (ThermoFisher). Libraries were sequenced to a depth of 1 × 10^8^ paired end reads (2 × 150 bp) per sample on the Illumina NextSeq platform. Sequencing reads were aligned to the hg19 reference genome and analyzed using Ambry's Bioinformatic Pipeline for alternative splicing events and differentially expressed genes.

### Bioinformatics analysis

Paired-end RNA-seq reads (2 × 250 bp) and Sanger sequencing reads (~1,100 bp) were first aligned to the hg19 human reference genome. For Sanger reads, GMAP aligner (version 2016-04-04) was used with default parameters to perform single transcript alignment (STA) of very long reads. For CloneSeq reads, STAR aligner v2.5.2a was used with default parameters except the “outSAMtype” parameter was set to “BAM SortedByCoordinate.” The mapped reads were then analyzed by our customized Ambry Bioinformatics Pipeline (ABP) software to detect splicing events such as exon skipping, alternative 5′ donor site, alternative 3′ acceptor site, and intron retention (15). These events are detected by the pipeline, based on the alignments against the reference genome (Figure 1B): (1) exon skipping, if there is no reads align to one exon or several consecutive exons; (2) partial exon skipping, if there is no read alignment in one end of an exon; (3) partial intron inclusion, if there is alignment in one end of an intron; (4) intron retention, if there is alignment in a whole intron; (5) cryptic exon, if there is alignment in the middle of an intron and no alignment in the rest of the intron. Schematic representations of these splicing events are illustrated in Figure 1B. To quantify splicing events, we calculated the percentage of alternative splicing event against a given transcript/isoform: percent of alternative splicing event = (number of reads supporting alternative splicing event)/(number of all reads in the region covering alternative splicing event). To filter out noise caused by sequencing and alignment errors, or due to the expression of ultra-rare isoforms, the splicing events with “number of reads supporting alternative splicing event” <20, or “number of all reads in the region covering splicing event” <50, or “percent of splicing event” <2.5% were filtered out. HGVS nomenclature values were approximate for intron retention and alternative splicing site events due to differences in alignments based on NGS reads.

## Results

### Quantitative and qualitative RNA analysis of the variant *BRCA1* c.5467+5G>T

**Figure 2:**
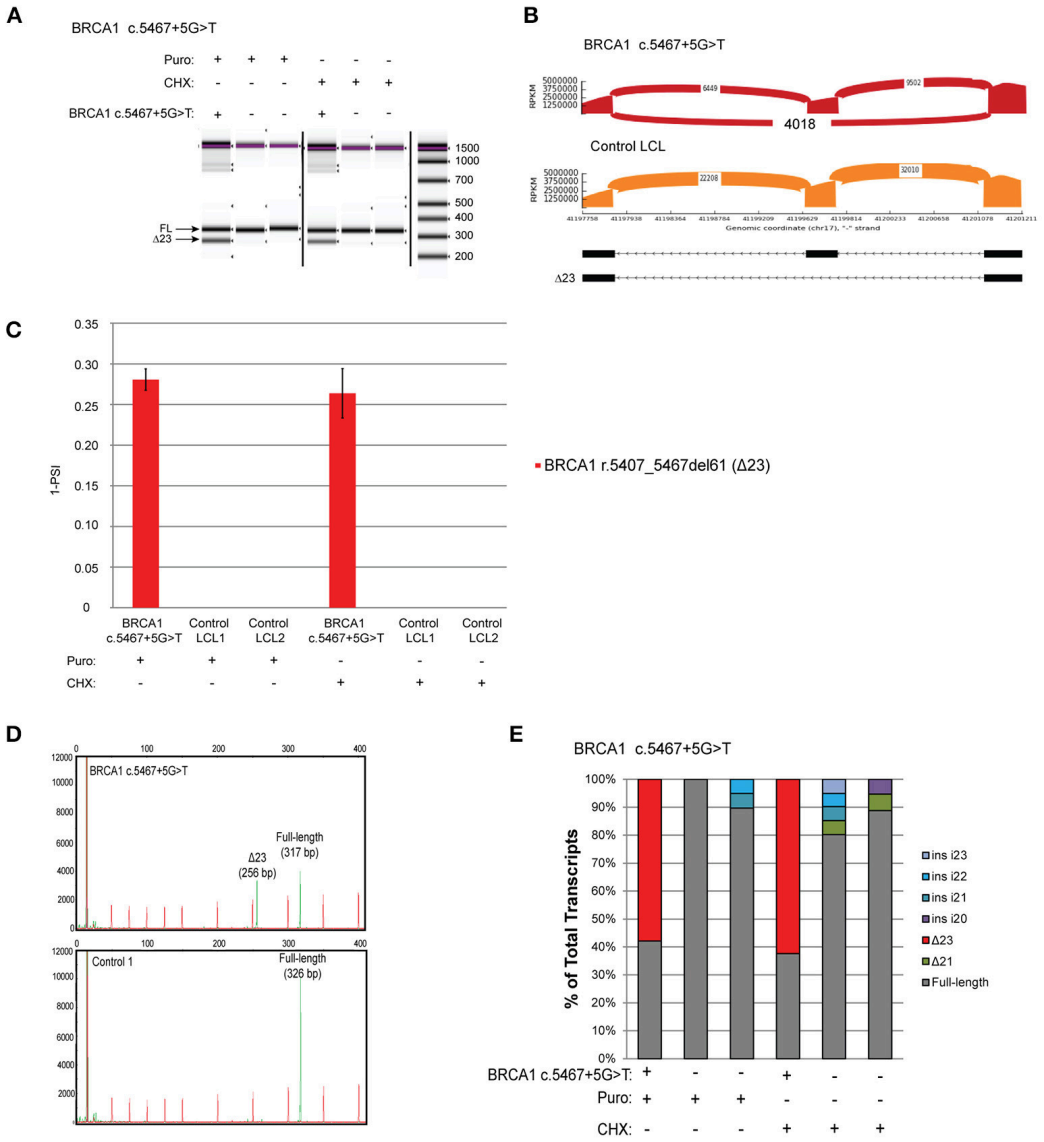
Quantitative and Qualitative RNA Analysis of the variant *BRCA1* c.5467+5G>T **(A)** Digital electrophoresis analysis of the RT-PCR performed on the *BRCA1* c.5467+5G>T carrier LCL and control LCLs treated with puro or CHX. **(B)** Sashimi plots of CloneSeq performed in the *BRCA1* c.5467+5G>T carrier LCL and control LCL. **(C)** Relative quantification of CloneSeq results shown as “percent spliced in index” (PSI). **(D)** Capillary electrophoresis analysis of the RT-PCR performed in the *BRCA1* c.5467+5G>T carrier LCL and control LCL. **(E)** RT-PCR products were cloned and individual colonies were selected for Sanger sequencing. Median relative frequency of each detected transcript is graphed (*n* = 3 biological replicates).

The variant *BRCA1* c.5467+5G>T, which impairs the native donor splice site of *BRCA1* exon 23, has been described to result in skipping of exon 23 (Δ23) (8). This variant is currently classified as VUS by ENIGMA (class 3). LCLs were obtained from carriers of the variant and 2 controls, and reverse-transcriptase PCR (RT-PCR) was performed using the conditions recommended by Whiley et al. including the use of the same primers, reverse transcriptase, and NMD inhibitors puromycin (puro) and cycloheximide (CHX) (8). Skipping of exon 23 (Δ23) was clearly detected by digital electrophoresis of the RT-PCR products in the *BRCA1* c.5467+5G>T carrier's LCL, but not in two control LCLs treated with NMD inhibitors (Figure 2A). RT-PCR products were cloned and CloneSeq was performed on these samples for sequence characterization and quantification. The sequence and absolute number of reads observed in the carrier and control cell lines are shown using Sashimi plots (Figure 2B). Sashimi plots provide a quantitative visualization of aligned MPS reads that enables quantitative comparison of exon usage across samples (16). A total of 4,018 reads supporting exon 23 skipping (r.5407_5467del61) were detected in the *BRCA1* c.5467+5G>T LCL, whereas none was detected in the control LCL (Figure 2B). Abnormal transcripts levels were then measured as a “percent spliced in index” (PSI) (Figure 2C). PSI demonstrates the ratio between reads including or excluding exons, indicating how efficiently sequences of interest are spliced into transcripts (17). This analysis indicated that ~25% of transcripts expressed by the *BRCA1* c.5467+5G>T LCL contains skipping of exon 23, whereas skipping of exon 23 was not detected in negative control LCLs (Figure 2C).

To validate the CloneSeq results we performed, in the same set of samples, the mRNA splicing assays recommended by the members of the ENIGMA consortium (8), including capillary electrophoresis (Figure 2D), and Sanger sequencing of subcloned transcripts (Figure 2E). Capillary electrophoresis clearly detected Δ23 in the *BRCA1* c.5467+5G>T LCL, in addition to the full-length WT transcript (Figure 2D). Sanger sequencing also detected Δ23 exclusively in the *BRCA1* c.5467+5G>T carrier's LCL (Figure 2E).

### CloneSeq characterization of the pathogenic alteration *BRCA1* c.135-1G>T

**Figure 3:**
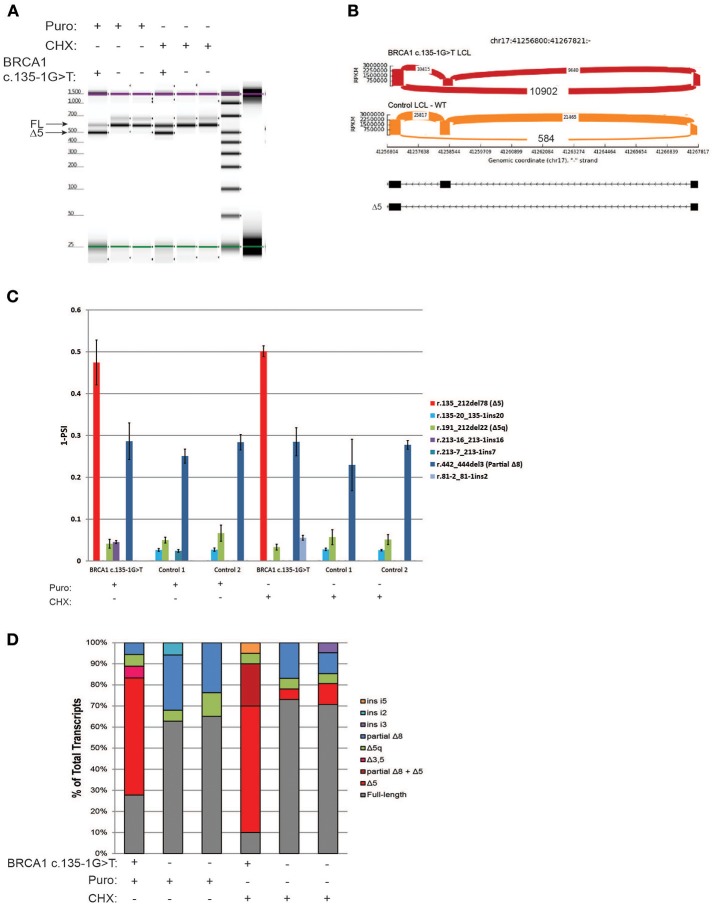
CloneSeq characterization of the pathogenic alteration *BRCA1* c.135-1G>T **(A)** Digital electrophoresis analysis of the RT-PCR performed on the *BRCA1* c.135-1G>T carrier LCL and control LCLs treated with puro or CHX. **(B)** Sashimi plots of CloneSeq performed in the *BRCA1* c.135-1G>T carrier LCL and control LCL. **(C)** Relative quantification of CloneSeq results shown as “percent spliced in index” (PSI). **(D)** RT-PCR products were cloned and individual colonies were selected for Sanger sequencing. Median relative frequency of each detected transcript is graphed (*n* = 3 biological replicates).

The variant *BRCA1* c.135-1G>T, which impairs the native acceptor site of *BRCA1* exon 5, has been associated with multiple splicing isoforms (8, 18), including an abundant transcript with skipping of exon 5 (Δ5). This variant is currently classified as pathogenic by ENIGMA (class 5). RT-PCR for the *BRCA1* c.135-1G>T carrier's LCL and control LCLs was performed following ENIGMA recommendations and analyzed by digital electrophoresis, which detected a band consistent in size with Δ5 (Figure 3A). RT-PCR products were cloned and CloneSeq performed, and the sequence and absolute number of reads observed in the carrier and control cell lines are shown using Sashimi plots (Figure 3B). A total of 10,902 reads supporting exon 5 skipping (r.135_212del78) were detected in the *BRCA1* c.135-1G>T LCL, whereas only 584 reads were detected in the control LCL (Figure 3B). Quantification of splicing events indicated that the *BRCA1* c.135-1G>T LCL expresses ~50% of transcripts with skipping of exon 5, whereas LCL negative controls have negligible levels of Δ5 (Figure 3C). Individual colonies were selected for transcript confirmation by Sanger sequencing, and the Δ5 transcript was the most abundant abnormal transcripts in the *BRCA1* c.135-1G>T LCL (Figure 3D).

### CloneSeq characterization of the pathogenic alteration *BRCA2* c.8632+1G>A

**Figure 4:**
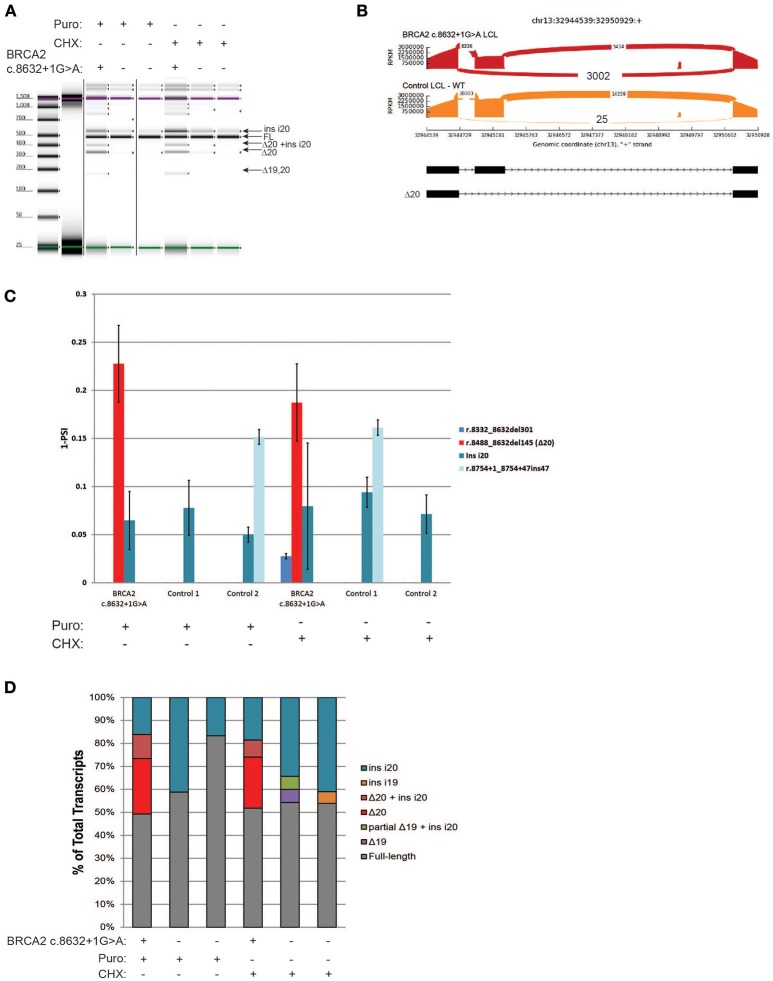
CloneSeq characterization of the pathogenic alteration *BRCA2* c.8632+1G>A **(A)** Digital electrophoresis analysis of the RT-PCR performed on the *BRCA2* c.8632+1G>A carrier LCL and control LCLs treated with puro or CHX. **(B)** Sashimi plots of CloneSeq performed in the *BRCA2* c.8632+1G>A carrier LCL and control LCL. **(C)** Relative quantification of CloneSeq results shown as “percent spliced in index” (PSI). **(D)** RT-PCR products were cloned and individual colonies were selected for Sanger sequencing. Median relative frequency of each detected transcript is graphed (*n* = 3 biological replicates).

*BRCA2* c.8632+1G>A, located at intron 20 of *BRCA2*, was shown to result in skipping of exon 20 (Δ20) (8), and is currently classified by ENIGMA as a pathogenic alteration (class 5). RT-PCR of the *BRCA2* c.8632+1G>A carrier's LCL and control LCLs was performed following ENIGMA recommendations. Digital electrophoresis analysis of the RT-PCR products detected several alternative transcripts, in addition to the full-length mRNA (Figure 4A). RT-PCR products were cloned and CloneSeq was performed. The sequence and absolute number of reads observed are shown in Sashimi plots for the carrier and control cell lines demonstrating a total of 3,002 reads supporting exon 20 skipping (r.8488_8632del145) in the *BRCA2* c.8632+1G>A LCL, whereas only 25 reads supporting Δ20 were detected in the control LCL (Figure 4B). Quantification of splicing events indicated that ~20% of the transcripts expressed by the *BRCA2* c.8632+1G>A LCL have skipping of exon 20, whereas LCL negative controls have negligible levels of Δ20 (Figure 4C). Several alternative spliced transcripts, detected both in the *BRCA2* c.8632+1G>A LCL and controls, were also identified (Figure 4C). Individual colonies were selected for transcript confirmation by Sanger sequencing, which identified the most abundant abnormal transcript Δ20 in the *BRCA2* c.8632+1G>A LCL, in addition to confirming other minor alternative isoforms detected by CloneSeq (Figure 4D).

### Characterization of the variant *BRCA2* c.9501+3A>T in LCLs and blood samples

**Figure 5:**
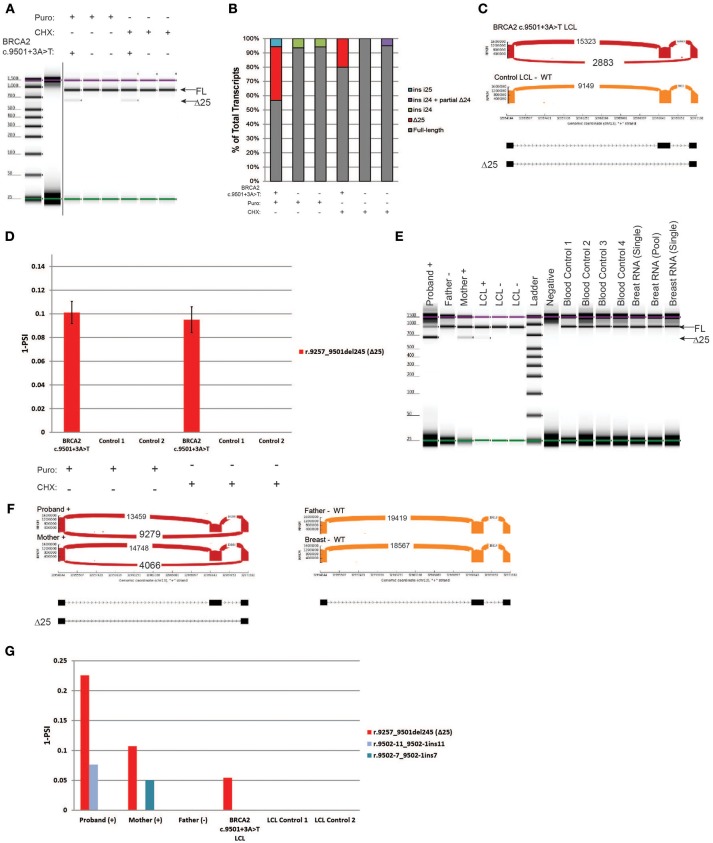
Characterization of the variant *BRCA2* c.9501+3A>T in LCLs and patient samples **(A)** Digital electrophoresis analysis of the RT-PCR performed on the *BRCA2* c.9501+3A>T carrier LCL and control LCLs treated with puro or CHX. **(B)** RT-PCR products were cloned and individual colonies were selected for Sanger sequencing. Median relative frequency of each detected transcript is graphed (*n* = 3 biological replicates). **(C)** Sashimi plots of CloneSeq performed in the *BRCA2* c.9501+3A>T carrier LCL and control LCL. **(D)** Relative quantification (PSI) of CloneSeq results obtained from the *BRCA2* c.9501+3A>T carrier LCL and control LCL. **(E)** Digital electrophoresis analysis of the RT-PCR performed on RNA obtained from the blood of *BRCA2* c.9501+3A>T carriers and control samples. **(F)** Sashimi plots of CloneSeq performed on RNA obtained from the blood of the *BRCA2* c.9501+3A>T carriers (proband and mother) and control individuals negative for the alteration (father and control breast tissue). **(G)** Relative quantification (PSI) of CloneSeq results obtained from *BRCA2* c.9501+3A>T carriers' blood, *BRCA2* c.9501+3A>T carrier LCL (LCL+), and negative controls (Father's blood and control LCLs).

*BRCA2* c.9501+3A>T is located in the native donor site of intron 25. This variant was reported to result in low levels of skipping of exon 25 (Δ25), and it is currently classified as benign (class 1) by ENIGMA (8). RT-PCR was performed on the carrier's LCL and on the control cells. Digital electrophoresis identified a minor band consistent with the size of Δ25 in the *BRCA2* c.9501+3A>T LCL that was not detected in negative controls (Figure 5A). RT-PCR products were cloned, and individual colonies were selected for Sanger sequencing, which detected Δ25 (r.9257_9501del245) in the *BRCA2* c.9501+3A>T LCL, in addition to low levels of other alternatively spliced transcripts in controls (Figure 5B). To more accurately quantify the relative abundance of Δ25, we performed CloneSeq. The assay detected a total of 2,883 reads supporting exon 25 skipping in the *BRCA2* c.9501+3A>T LCL, whereas no reads supporting skipping of this exon were detected in the control LCL (Figure 5C). The reads supporting Δ25 were ~10% of the total splicing events detected in the *BRCA2* c.9501+3A>T LCL (Figure 5D).

Subsequently, we compared CloneSeq LCL results with an analysis of RNA isolated from the blood cells of carriers of the *BRCA2* c.9501+3A>T alteration. RT-PCR performed on RNA from individuals that are heterozygous for *BRCA2* c.9501+3A>T (proband and mother) and negative controls (father, LCL-, normal breast RNA, and normal blood controls) detected Δ25 only in the positive samples (Figure 5E). CloneSeq was performed to quantify Δ25 in these samples, which detected 9,279 and 4,066 splicing events supporting Δ25 in the proband and mother's samples respectively, while none were detected in the negative controls (Figure 5F). Interestingly, the percentage of Δ25 transcripts varies among different carriers. For the proband, Δ25 represents ~20% of splicing events, while it accounted for ~10% in the mother's sample (Figure 5G), ~5% in the LCL+ *BRCA2* c.9501+3A>T without NMD inhibition (Figure 5G), and ~10% of splicing events when the LCL+ is treated with inhibitors (Figure 5D).

### Characterization of a novel variant, *BRCA1* c.5152+5G>T

**Figure 6:**
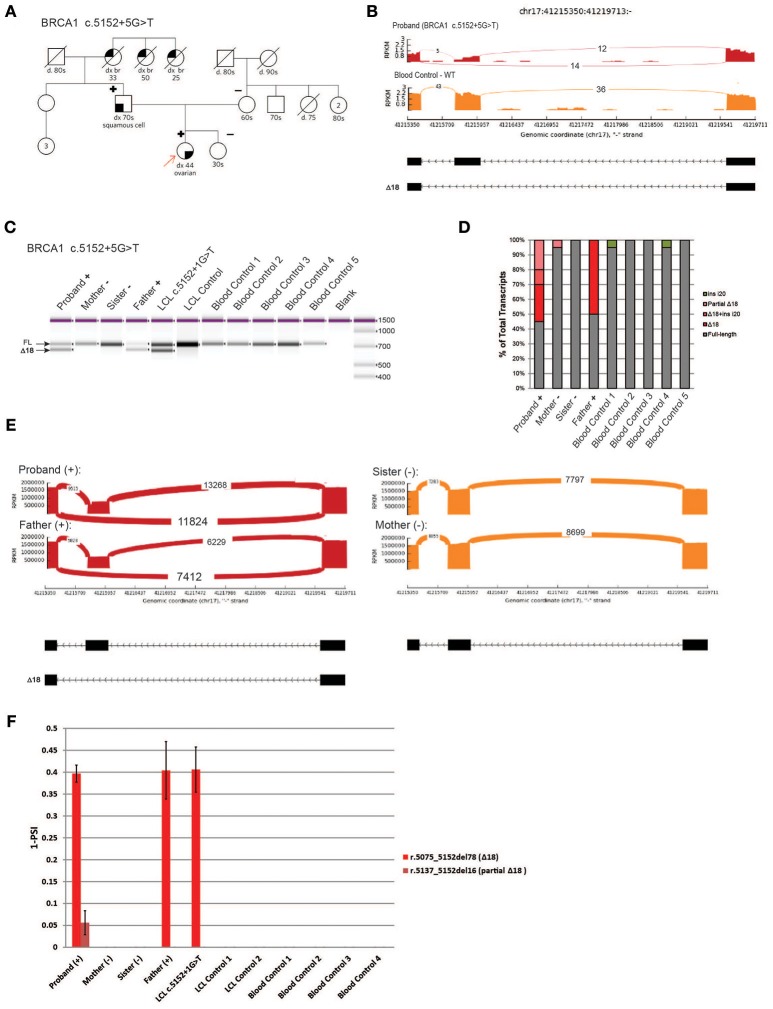
Characterization of a novel variant, *BRCA1* c.5152+5G>T **(A)** Pedigree of HBOC family carrying the variant *BRCA1* c.5152+5G>T. **(B)** Sashimi plots of whole transcriptome sequencing performed on RNA obtained from the blood of a *BRCA1* c.5152+5G>T carrier and control sample. **(C)** Digital electrophoresis analysis of the RT-PCR performed in RNA obtained from blood of *BRCA1* c.5152+5G>T carriers (proband and father), *BRCA1* c.5152+1G>T carrier LCL, and control samples negative for the alteration (RNA obtained from the mother and sister's blood, negative LCL, negative unrelated blood controls). **(D)** RT-PCR products were cloned and individual colonies were selected for Sanger sequencing. Median relative frequency of each detected transcript is graphed (*n* = 3 biological replicates). **(E)** Sashimi plots of CloneSeq performed in RNA obtained from blood of the *BRCA1* c.5152+5G>T carriers (proband and father) and control individuals negative for the alteration (sister and mother). **(F)** Relative quantification (PSI) of CloneSeq results obtained from *BRCA1* c.5152+5G>T carriers' blood (proband and father), *BRCA1* c.5152+1G>T carrier LCL, and several controls negative for the alteration (mother and sister, control LCL, and unrelated blood controls).

We next analyzed a novel uncharacterized VUS, *BRCA1* c.5152+5G>T, identified in a HBOC family (Figure 6A). This rare variant is located in the donor splice site of intron 18 at a highly conserved position, and was predicted by several splicing *in silico* programs to abolish the splice site (data not shown). We obtained blood samples from patients that are heterozygous for *BRCA1* c.5152+5G>T (proband and father) as well as samples from negative individuals (proband's mother and sister). To characterize the variant we performed whole transcriptome sequencing (WTS) on the proband and control blood samples. WTS detected 14 reads supporting skipping of exon 18 (Δ18, r.5075_5152del78) and 12 reads supporting the WT transcript in the proband (Figure 6B, top). In the control blood sample, only WT reads (*n* = 36) were detected (Figure 6B, bottom). Primers were designed in the flanking exons and RT-PCR was performed. Digital electrophoresis analysis of RT-PCR products identified a band corresponding to Δ18, exclusively in samples from the heterozygous carriers (Figure 6C). Sanger sequencing of subcloned transcripts was then performed, which confirmed Δ18 sequence (Figure 6D), indicating it results in in-frame skipping of the important BRCT functional domain of BRCA1 (19). Finally, using CloneSeq, we detected and quantified the Δ18 transcript in heterozygous individuals, which was undetectable in non-carriers (Figure 6E). CloneSeq detected 11,824 reads supporting Δ18 (r. c.5075_5152del78) and 13,268 reads supporting WT transcript in the proband (Figure 6E, left top). In the proband's father, CloneSeq detected 7,412 reads supporting Δ18, and 6,229 reads supporting WT transcript (Figure 6E, left bottom). Quantitatively, we found heterozygous individuals to express ~40% of Δ18 transcripts (Figure 6F). Of note, analysis of the LCL harboring the pathogenic alteration *BRCA1* c.5152+1G>T, affecting the same donor splice site as *BRCA1* c.5152+5G>T, also led to similar expression of the abnormal transcript Δ18 (Figures 6C,F). Altogether, these data were used to reclassify *BRCA1* c.5152+5G>T from VUS to likely pathogenic, and therefore a clinically actionable alteration.

## Discussion

Genetic testing for HBOC is becoming increasingly widespread in the era of precision medicine (20, 21). The implementation of next-generation sequencing has resulted in an explosion of genetic data, and germline variants with unknown function are regularly detected by clinical diagnostic laboratories (22). In particular, VUS in clinically actionable genes, such as the HBOC susceptibility genes *BRCA1* and *BRCA2*, pose a quandary to medical providers and patients (23–25). A specific challenge is the large percentage of VUS in the *BRCA1* and *BRCA2* genes predicted to affect splicing by *in silico* tools, but lack RNA evidence (26). In part, this is due to the scarcity of high-throughput assays designed to interrogate the impact of variants on splicing. The American College of Medical Genetics and Genomics and the Association for Molecular Pathology (ACMG/AMP) guidelines for the interpretation of germline sequence variants recommends the use of multiple types of evidence for classifying variants identified by DNA genetic testing, such as functional evidence, allele frequency data, computational and *in silico* predictions, and phenotype/family history (1). Therefore, RNA testing is critical to perform a comprehensive interpretation of sequence variants predicted to affect splicing. With this in mind, there are several RNA assays that have been used to characterize splicing alterations, each assay possessing its own advantages and drawbacks. These include the use of hybrid minigenes for the characterization of candidate splicing variants (27, 28). Given that a hybrid minigene assay analyzes the splicing outcome of a single allele, it is a great toll to evaluate allele-specific expression, i.e., the demonstration that the variant allele produces highly expressed abnormal transcripts predicted to induce NMD or to disrupt clinically important residues, an important step for the classification of splicing variants as pathogenic (10, 11). A caveat of this technique is the requirement of constructing and using artificial vectors (which are not available to most commercial diagnostic laboratories), and the need to test cell lines instead of samples derived from the patient being evaluated. The assays recommended by the ENIGMA consortium to characterize *BRCA1* and *BRCA2* transcripts include capillary electrophoresis and Sanger sequencing of subcloned transcripts, (8, 29). These assays are accessible to most laboratories, can be performed in patient samples, do not require the use of expression vectors, and can perform qualitative characterization of splicing variants; however, these assays only provide semi-quantitative data and lack the throughput required to analyze a large amount of variants (30, 31). Alternatively, there are quantitative approaches, including real-time and digital PCR, that provide robust and reliable quantitative data (29, 32), but cannot perform qualitative analysis (i.e., these assays are unable to reveal the precise transcript sequence). Here we describe a novel RNA MPS method, CloneSeq, and demonstrated that this technique is capable of performing reliable high-throughput quantitative and qualitative analysis of splicing variants, a necessary feature to obtain evidence for the large number of alterations predicted to affect splicing in HBOC genes.

Using CloneSeq coupled to our custom ABP bioinformatics analysis we were able to detect all major splicing aberrations described by the ENIGMA consortium in four well-characterized LCLs (8), with the advantage of obtaining absolute and relative quantification of the expressed transcripts (Figures 2–5). CloneSeq proficiently detected abnormal transcripts, as well as less abundant alternative splicing events, as it analyzed thousands of cloned reads. We validated the CloneSeq results using digital and capillary electrophoresis and Sanger sequencing of subcloned transcripts. Digital and capillary electrophoresis identified the abundant abnormally spliced transcripts detected by CloneSeq, but these methods were incapable of providing proper quantification of transcript levels due to their semi-quantitative nature. Additionally, sequencing was needed to confidently identify the exact splicing event detected by digital and capillary electrophoresis. Both CloneSeq and Sanger sequencing were able to precisely determine the sequence of the transcripts, however, CloneSeq performed sequencing of tens of thousands of transcripts (average number of *BRCA1* or *BRCA2* mapped reads *per* sample tested was 24,803). In comparison, Sanger sequencing is limited to low-throughput sequencing of colonies, each containing a single subcloned transcript (median = 56 clones sequenced *per* LCL). As examples of its high analytical sensitivity, CloneSeq was able to detect the splicing isoforms induced by the variants *BRCA1* c.135-1G>T and *BRCA2* c.8632+1G>A, and rare alternative spliced isoforms previously reported by ENIGMA (Figures 3, 4). Ultimately, CloneSeq's targeted ultra-deep *locus* sequencing of *BRCA1* or *BRCA2* proved to be a fundamental feature for the bioinformatics qualitative and quantitative characterization of splicing alterations in these genes.

In addition to LCLs, we analyzed RNA extracted from blood samples obtained from variant carriers and controls. For the previously characterized *BRCA2* c.9501+3A>T benign variant (class 1), we were able to compare LCL RNA data with RNA data from the blood of heterozygous *BRCA2* c.9501+3A>T carriers. Similar to Sanger sequencing, CloneSeq detected the major abnormal splicing event associated with this variant, skipping of exon 25, both in LCLs and in carriers' blood RNA. Because quantification of abnormally and alternatively spliced transcripts is fundamental to predict pathogenicity (10, 11, 29), we quantified the impact of this benign variant on splicing levels. The percentage of skipped exon 25 identified in different *BRCA2* c.9501+3A>T carriers ranged from ~20 to ~10% of total splicing events, suggesting that an alteration resulting in less than ~20% of abnormal splicing is clinically benign. However, it is important to note, this is an indirect measurement of each allele's expression, since we were unable to perform allele-specific expression in the individuals we tested due to the lack of informative variants in the coding sequence of *BRCA2* in the respective samples. In order to mitigate this caveat, we ran a series of normal blood and tissue controls to identify and differentiate any physiologic alternatively spliced isoform from abnormal transcripts identified in the variant carriers (Figures 5E–G). The CloneSeq results are also in agreement with a minigene single-allele analysis of this alteration that reported ~13% of Δ25 is induced by the variant *BRCA2* c.9501+3A>T (27). By quantifying the impact that variants with benign clinical behavior have on splicing, CloneSeq could be used in the future to identify a splicing threshold that must be reached by abnormal transcripts in order to classify a *BRCA1* or *BRCA2* alteration as pathogenic.

Lastly, we analyzed blood samples obtained from a HBOC family carrying a novel uncharacterized VUS, *BRCA1* c.5152+5G>T. To characterize the VUS we performed whole transcriptome sequencing in the proband and control blood samples. WTS detected 14 reads supporting skipping of exon 18, and 12 reads supporting the WT transcript in the proband. Using CloneSeq, we were able to detect in the proband 11,824 reads supporting skipping of exon 18 and 13,268 reads supporting the WT transcript. Comparatively, the number of reads detected by WTS vs. CloneSeq highlights the higher analytical sensitivity of the later. This supports the notion that WTS can provide biased results due to low detection yields and other technical limitations (33, 34). On the other hand, CloneSeq provided the sufficient sequencing depth necessary for transcript characterization and quantification, which proved to be indispensable to reclassify the VUS *BRCA1* c.5152+5G>T as a clinically actionable likely pathogenic alteration.

Massively parallel sequencing is revolutionizing cancer genetics by enabling the detection and characterization of sequence variants at unprecedented scale and speed. For example, depending on the technology and protocol used, the number of individuals tested *per* variant, and the number of controls tested, CloneSeq can concomitantly perform analysis of multiple variants in a single MPS run (up to 96 samples). From the initial RNA extraction steps to the final bioinformatics analysis, the protocol described here can analyze multiple samples in less than 10 days (Figure 1A). Even though NGS technologies have evolved quickly over the past decade, leading to a substantial decrease in the cost per megabase (30), the cost of NGS assays may still pose challenges to laboratories with low throughput (31). However, implementation of automated steps and the development of innovative sequencing technologies could reduce the cost and time-frame of CloneSeq even further in the near future. Besides laboratory costs, one important issue to consider is the impact RNA genetic testing has on variant classification. One of the caveats of DNA MPS multi-gene testing is the high rate of VUS results (35). Since RNA genetic testing has the potential to reduce VUS rates, future research should investigate the broader impact these tests have on the overall clinical management of patients identified with germline variants in *BRCA1* and *BRCA2*.

## Conclusion

CloneSeq is an alternative to the current splicing assays recommended by the ENIGMA consortium. Due to its high-throughput format, quantitative and qualitative abilities, and high analytical sensitivity, CloneSeq has the potential to improve the interpretation of splicing sequence variants detected by HBOC clinical genetic tests. The enhanced classification of these germline variants as either disease-causing or neutral is fundamental to offering preventive pre-symptomatic measures or targeted treatment to the correct individuals.

## Author contributions

SF-K and RK drove the development of the intellectual concepts, performed analyses, interpreted data, and wrote the manuscript. VH, JH, SL, and TL performed experiments, generated data, and wrote the manuscript. SW, HV, DX, BL, and H-ML developed and ran the bioinformatics pipeline, and wrote the manuscript. GH, SN, and DT collected data, samples, and wrote the manuscript. PG, BT, and AE contributed to manuscript writing and to experimental design.

### Conflict of interest statement

All authors, with the exception of SN and DT, were employees of Ambry Genetics when they were engaged with this project. The remaining authors declare that the research was conducted in the absence of any commercial or financial relationships that could be construed as a potential conflict of interest.
